# Complement-mediated HUS revisited: evolving insights into pathophysiology, diagnosis, and treatment

**DOI:** 10.3389/fimmu.2025.1621317

**Published:** 2026-01-14

**Authors:** Ruah Alyamany, Ann M Moyer, Maria Alice V. Willrich, Meera Sridharan

**Affiliations:** 1 Division of Hematology, Department of Internal Medicine, Mayo Clinic, Rochester, MN, United States; 2 Department of Laboratory Medicine and Pathology, Mayo Clinic, Rochester, MN, United States

**Keywords:** aHUS, TMA, complement, thrombotic microangiopathy, hemolytic uremic syndrome

## Abstract

Complement-mediated hemolytic uremic syndrome (CM-HUS), commonly referred to as atypical HUS, is a rare thrombotic microangiopathy caused by uncontrolled activation of the alternative complement pathway, typically triggered by a “two-hit” mechanism. It is characterized by microangiopathic hemolytic anemia, thrombocytopenia, and end-organ damage, most commonly affecting the kidneys. While our understanding of the complement system has advanced significantly, CM-HUS remains a complex, heterogeneous disorder influenced by a spectrum of genetic variants, risk haplotypes, and acquired factors such as anti-factor H autoantibodies. This review highlights the current knowledge of CM-HUS pathogenesis, focusing on genetic variants in regulatory and activating proteins of the complement system. We also discuss the diagnostic complexity posed by incomplete penetrance, overlapping phenotypes, and limitations of genetic and functional assays. Emerging ex-vivo assays and complement biomarkers are explored as tools for refining diagnosis and risk stratification. The use of complement inhibitors such as eculizumab and ravulizumab has significantly improved renal outcomes and survival. This review provides a comprehensive, clinically grounded update on the genetics, pathophysiology, diagnostics, and therapeutic considerations in CM-HUS, aiming to provide clinicians and researchers with a deeper understanding of this complex, complement-driven disease.

## Graphical Abstract

**Figure:**
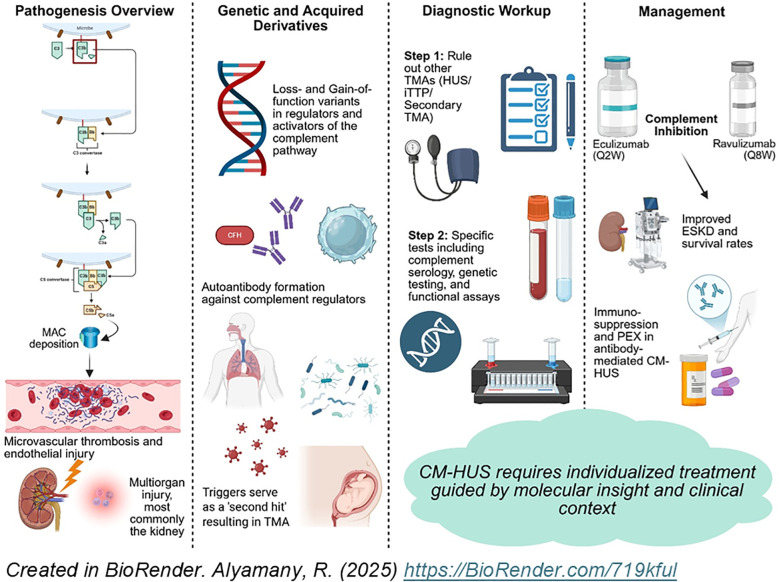
Created in BioRender. Alyamany, R. (2025) https://BioRender.com/719kful.

## 1 Introduction

Thrombotic microangiopathies (TMAs) are a heterogenous group of disorders characterized by thrombocytopenia, microangiopathic hemolytic anemia (MAHA), and end-organ damage. Atypical hemolytic uremic syndrome (aHUS), more precisely termed complement-mediated hemolytic uremic syndrome (CM-HUS), is a rare, incidence ranging between 0.23 – 1.9 per million population per year, but potentially life-threatening condition (1). Incidence and prevalence vary based on region and age (1).Unlike Shiga toxin-associated HUS and immune thrombotic thrombocytopenic purpura (iTTP), CM-HUS is primarily driven by dysregulation of the alternative complement pathway, resulting in uncontrolled activation, excessive inflammation, and endothelial injury.

In this review, we summarize current knowledge on the role of complement proteins and autoantibodies in the pathogenesis, diagnosis, and treatment of CM-HUS.

### 1.1 Case 1

An 8-year-old female presented with dark brown colored urine and yellowing of the skin. Two weeks prior, she had experienced abdominal pain and a single episode of non-bloody diarrhea. Two days before presentation, she developed emesis, fever, cough, and rhinorrhea. Laboratory results at presentation showed anemia (hemoglobin 6.5 g/dL), thrombocytopenia (platelets 45 x 10^9^/L), and elevated creatinine (3.99 mg/dL). LDH was elevated at 723 U/L, and haptoglobin was <14 mg/dL. Peripheral blood smear revealed schistocytes. She was diagnosed with hemolytic uremic syndrome. Stool testing for Shiga toxin was negative. ADAMTS13 activity was 88%. She had no other medical diagnoses. CM-HUS was suspected. She initiated dialysis and was urgently started on eculizumab. She received vaccination against *Neisseria meningitidis*. Complement genetic testing revealed a homozygous deletion of *CFHR3-CFHR1*. Additional testing showed a deletion between the *CFH* and *CFHR* region, which supported abnormal CFH function. Serologic testing during hospitalization was positive for *Salmonella typhi* antibodies, suggesting a possible infectious trigger for the acute episode.

Following initiation of eculizumab, her thrombocytopenia and hemolytic anemia improved. Factor H autoantibody testing was positive at 92.1 U/mL (normal <15.8). She was started on mycophenolate mofetil and three months later, Factor H antibodies became undetectable. The decision was made to continue eculizumab.

### 1.2 Case 2

A 34-year-old female presented with headaches, nausea, abdominal discomfort, and non-bloody diarrhea. A week earlier, she had eaten at a restaurant. On presentation, she was found to have renal injury, thrombocytopenia, and laboratory features consistent with MAHA. Her PLASMIC score was 5. She received plasma exchange for five days, with improvement in platelet count; however, due to recurrent thrombocytopenia upon cessation of plasma exchange and persistent renal dysfunction, eculizumab was started. ADAMTS13 activity was 90%. Stool testing for Shiga toxin was negative. Complement serologies demonstrated an elevated soluble membrane attack complex (sMAC), low C3, and normal C4 concentration. Factor H antibody testing was negative. One month after starting eculizumab, she achieved hematologic and renal remission. Complement genetic testing demonstrated no pathogenic variants. Six months after initiating therapy, eculizumab was discontinued.

## 2 Evaluation of complement-mediated HUS

*The cases demonstrate two patients with a shared underlying disease process but distinct clinical presentation and age at onset*.

Patients with CM-HUS typically present with MAHA, thrombocytopenia, and end-organ damage, often manifesting as acute kidney injury (AKI). Compared to other forms of TMA, CM-HUS is characterized by more severe renal involvement, often progressing to end-stage kidney disease (ESKD) if untreated. Unlike iTTP, CM-HUS is less responsive to plasma exchange, and renal deterioration may continue despite intervention (2, 3). Hypertension is a common clinical feature, and distinguishing CM-HUS from hypertensive emergency can be challenging.

Extra-renal manifestations, including neurological symptoms such as seizures, confusion, and encephalopathy, as well as gastrointestinal involvement such as hepatotoxicity, pancreatitis, and gastrointestinal bleeding, are less common but have been reported (3).

*In both cases presented, patients had thrombocytopenia, MAHA, and end-organ damage in the form of kidney injury. Case 2 also exhibited extra-renal manifestations, including altered mental status.*

The diagnosis of CM-HUS is one of exclusion. The initial step involves ruling out other potential causes of TMA, including Shiga toxin-associated HUS, TTP (ruled out by demonstrating ADAMTS13 activity >10%), and TMAs secondary to conditions such as medications, malignant hypertension, autoimmune disease, transplant, or pregnancy. If no alternative etiology is identified, a working diagnosis of CM-HUS should be pursued and treatment with complement inhibitor therapy should be initiated. Laboratory evaluation aims to detect complement abnormalities that support the clinical suspicion of CM-HUS. Testing options include complement serology, autoantibody assessment, evaluation of complement activation on cell membranes, and complement genetic analysis (4).

Below we describe the complement abnormalities associated with CM-HUS and outline how laboratory tests evaluate these abnormalities to support the clinical diagnosis.

## 3 Complement abnormalities

**Figure 1:**
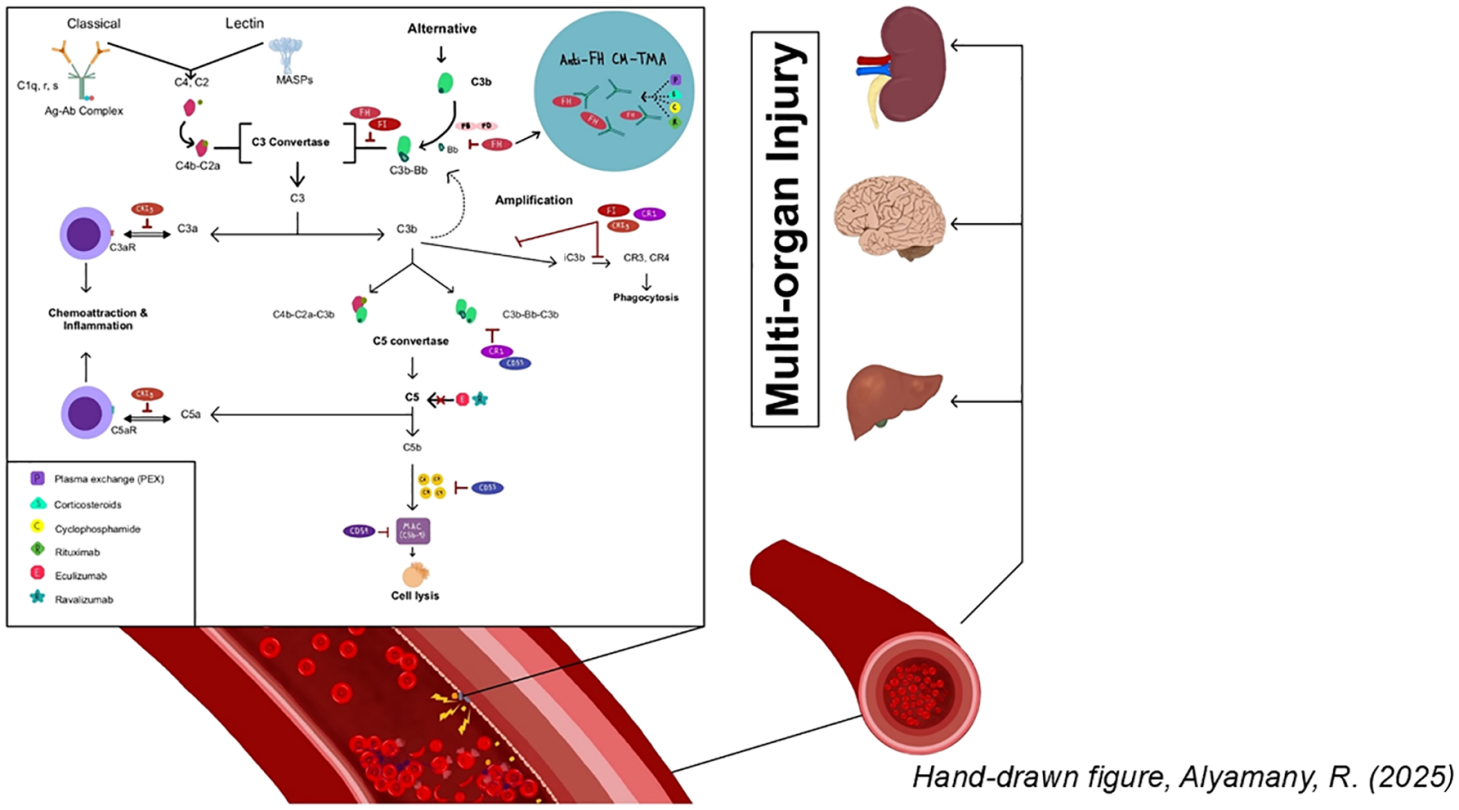
Dysregulation of the complement pathway and associated therapeutic targets in complement-mediated hemolytic uremic syndrome (CM-HUS).

The pathophysiologic mechanism underlying CM-HUS involves the dysregulation of the amplification loop of the alternative pathway leading to unregulated formation of the the membrane attack complex, and subsequent tissue injury (**Figure 1**). CM-HUS is a genetic disorder that may occur in either familial or sporadic forms. The first gene linked to CM-HUS, *CFH*, was identified in 1998. Since then, variants in several additional complement-related genes have been implicated. Most pathogenic variants are heterozygous, and approximately 20% of patients have a combination of mutations (5). A key challenge in CM-HUS diagnosis is that only 40-50% of affected individuals have identifiable pathogenic variants in complement-regulating proteins.

**Figure 2:**
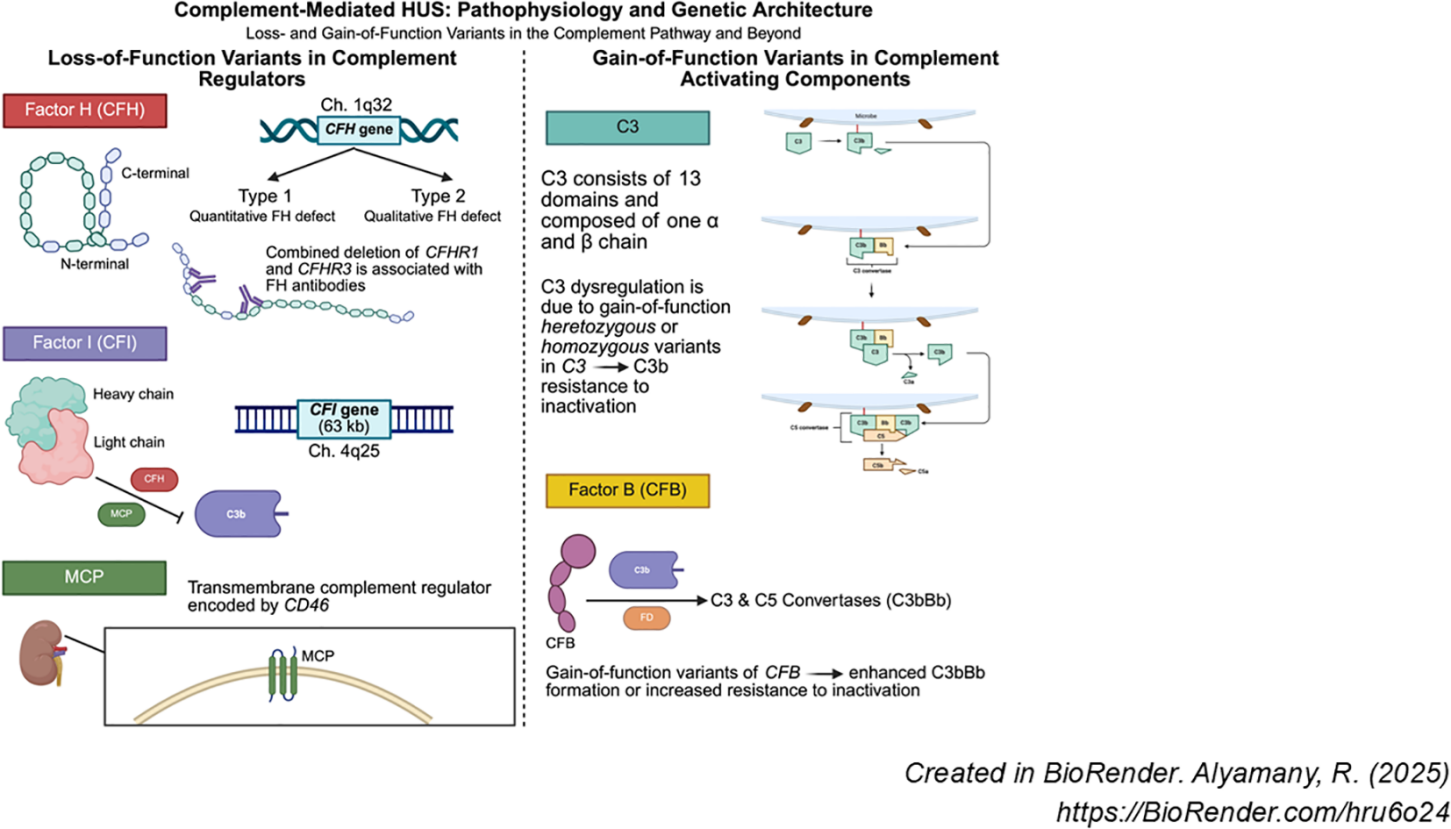
Pathophysiology and Genetic Architecture of CM-HUS. Created in BioRender. Alyamany, R. (2025) https://BioRender.com/hru6o24. .

Complement gene variants implicated in CM-HUS include gain-of-function variants in complement activators or loss-of-function variants in complement regulators (**Figure 2**). More than 120 variants in *CFH*, *CFI*, and CD46 *(MCP*) have been reported among patients with CM-HUS (6).

In addition to pathogenic complement gene variants, other genetic features that can modify risk are haplotypes, which are a specific combination of alleles at adjacent loci along the same chromosome that are inherited together as a unit (7). In CM-HUS, haplotypes on their own are not sufficiently pathogenic to cause disease on their own, but when they occur alongside pathogenic variants they can increase the risk of developing the disease or manifestations of the disease (ie. end stage renal disease) (8, 9).

CM-HUS is not a monogenic disorder as patients may carry multiple disease-associated variants or risk haplotypes. Although CM-HUS is considered a genetic disease, due to incomplete penetrance, the presence of genetic variants alone is insufficient to cause clinical TMA. Instead, at least two “hits” are generally required. The second hit typically involves an environmental trigger. In a cohort of 272 patients, a triggering event was identified in 70% of cases, most commonly diarrheal or gastrointestinal illnesses and upper respiratory tract infections (6). Pregnancy is another, though less common, trigger.

Depending on the genetic variant(s) identified, the risk of developing CM-HUS, as well as clinical manifestations and outcome, may vary (10, 11). For instance, individuals with *CFH* or *CFI* variants are more likely to present before one year of age (12). Below (**Table 1**), we summarize the current understanding of the most commonly implicated genes and proteins in CM-HUS.

**Table 1:** Complement Genes and Proteins in CM-HUS.

Protein (*Gene*)*	% of pathogenic gene variants in CM-HUS	Exons	Protein MW (kDa)	Target proteins	Main protein function
Loss-of-function variants in complement regulators					
Factor H (*CFH*)	15-30%	22	155	C3b, C3c, C3d, C3 (H_2_O), GAGs, heparin, FI, TM, CRP, DNA, annexinII, histones, fibronectin oligomerization	Negative regulator of the alternative pathway C3 convertase: Dissociates C3bBB complex, prevents FB binding to C3b and serves as a cofactor for FI for the inactivation of C3 to iC3b
MCP (*CD46)*	10-15%	14	4 iso forms	C3(H2O), C3b, C4b	Serves as a cofactor for FI for the cleavage of C3b on the cell surface
Factor I (*CFI*)	5-15%	13	88	FH, MCP, CR1, TM, C3b, C3 (H2O), C4b	Cleaves and inactivates C3b and C4b, using FH, MCP, CR1, and TM as cofactors
Gain-of-function variants in complement activating components					
C3 (*C3*)	2-10%	42	187	Many binding partners: FB, FH, properdin, C5, MCP, CR1, CR2, CR3, C3 is cleaved by the classical and alternative pathway C3 convertases to C3b and degraded by FI in the presence of cofactors (FH, MCP, CR1, C4Bp, TM)	Central Component of the complement system. Forms C3 and C5 convertases
Factor B (CFB)	0-3%	18	90	C3b, C3 (H2O), Factor D, CVF	Serine protease of the alternative pathway C3 convertase

Adapted from Roumenia et al. (10).

* KDIGO guidelines (93) utilize the following risk stratifcation when considering prophyalxis against CM-HUS recurrence in renal allografts; High risk: patients with pathogenic variants; Moderate risk: patients with isolated *CFI* variants, variants of unknown signficance, or no variant; Low risk: isolated *CD46* variants.

CR1, complement receptor 1; CR2, complement receptor 2; CR3, complement receptor 3; CVF, cobra venom factor; FB, factor B; FH, factor H; FI, factor I; GAG, glycosaminoglycans; MCP, membrane cofactor protein; TM, thrombomodulin.

### 3.1 Loss-of-function variants in complement regulators

#### 3.1.1 Complement Factor H and complement Factor H related proteins

Complement factor H (FH), the predominant fluid-phase regulator of the alternative pathway of complement, acts as a cofactor for the cleavage of C3b and accelerates the decay of C3 convertase (13). Factor H is a single-chain polypepetide glycoprotein composed of 20 short consensus repeats (SCRs). The N-terminal (SCR1–4) and C-terminal (SCR19–20) serve as C3b-binding regions. The N-terminal SCRs (1–4) regulate complement amplification by binding to C3b, accelerating decay of the alternative pathway C3 convertase (C3bBb), and serving as a cofactor for factor I-mediated proteolytic inactivation of C3b (14).

Experimental mapping has identified a potential third, lower-affinity C3b-binding site in the mid-region (around SCR12–14), but its physiologic relevance is less well established. Consistent with this, pathogenic *CFH* variants associated with CM-HUS cluster predominantly in the N-terminal regulatory domains and, especially, in the C-terminal host-recognition domains, whereas relatively few disease-associated variants have been described in SCR12–14 (15, 16).

The C-terminal domain of FH is implicated in recognizing ligands specific to host surfaces. The simultaneous binding of C3b and these ligands is what allows FH to protect only host surfaces. The C-terminal SCRs (19, 20) are critical for protecting host endothelial surfaces (17, 18). The functional significance of SCR 15–18 has not been formally established but it is thought to play a structural role by allowing FH to bend which allows it to bind to different sites on C3b (16, 19).

*CFH*, a member of the regulator of complement activation gene cluster on chromosome 1q32, encodes FH. Since *CFH* variants were first identified in patients with CM-HUS, numerous studies have confirmed the association, with CFH variants accounting for approximately 15 – 30% of pathogenic variants in CM-HUS (20, 21). Most *CFH* variants are heterozygous, resulting in missense changes within the C- terminal portion. Although rare, nonsense and frameshift *CFH* variants that cause null alleles and affect the expression of CFH (16, 22) have also been described. *CFH* variants may be classified into two types: Type 1 variants impair FH secretion or folding, resulting in low plasma concentration of FH; type 2 variants produce functionally defective FH that fails to bind its targets (e.g. C3b, heparin, endothelial surfaces) (23).

Prior to complement inhibitor therapy, end stage kidney disease (ESKD)-free survival depended on the type of *CFH* variant, with worse outcomes in Type 2 C-terminal *CFH* and hybrid variants compared to Type 1 *CFH* variants (23).

In addition to FH, the FH protein family includes: FHR-1, FHR-2, FHR-3, FHR-4A, and FHR-5. These proteins are encoded by five genes: *CFHR1*, *CFHR2*, *CFHR3*, *CFHR4*, and *CFHR5*, which are present as a cluster 3’ of *CFH*. FHR proteins share high sequence homology with FH. FHR1, FHR3, FHR4 and FHR5 can compete with FH and dysregulate complement activation (24).

The *CFH and CFHR1–5* genomic region is rich in repeated sequences involving exons of the *CFH* and *CFHR1–5* genes (25, 26). Due to the high homology, genomic rearrangements are common in this region and may result in deletions, duplications, and formation of hybrid genes (27–29). A common combined deletion of *CFHR1* and *CFHR3* is associated with the development of autoantibodies against FH and an increased risk of CM-HUS, as discussed below.

The *CFH-H3* risk haplotype is a specific combination of variants within *CFH* and its surrounding genes. This haplotype consists of four single nucleotide changes (NM_000186.3: c.-331C>T, c.1204C>T, c.2016A>G, c.2808G>T) and two reference nucleotides (c.184G, c.2237-543G).) Although variants that are part of the *CFH* -H3 risk haplotype are common in the general population, if an individual has the appropriate pathogenic genetic variant (s) and environmental triggers, the presence of the risk haplotype may lead to an increased risk to develop CM-HUS (e.g. the *CFH-H3* risk haplotype may increase the penetrance) (30).

#### 3.1.2 Complement factor I

Complement factor I (CFI) is a serine protease that inactivates C3b using CFH and MCP (encoded by *CD46*) as cofactors (31). CFI is composed of a heavy and light chain linked by a disulfide bond. The heavy chain includes four domains: a factor I domain, a scavenger receptor protein domain, and two LDL receptor domains. The light chain contains the serine protease domain. In the presence of cofactors (CR1, MCP, FH), CFI cleaves the α chains of C3b and C4b, leading to inactive iC3b, C3f, C4c, and C4d (32, 33).

*CFI* is located on chromosome 4q25 and spans approximately 63 kilobases (34). CFI variants are found in 5–15% of CM-HUS cases (35) and generally have a minor allele frequency (MAF) <0.001. Most disease-associated *CFI* mutations impair protein secretion; in cases where secretion is preserved, the resulting protein is functionally deficient (36). Approximately 30% of patients with *CFI* variants also harbor genetic variants, including risk haplotypes in other complement genes (i.e. *CFH-H3 or MCP _ggaac_*), known to represent susceptibility factors for CM-HUS (37). Due to the limited number of familial cases of *CFI*-mediated CM-HUS, low penetrance is suspected (35).

#### 3.1.3 Membrane cofactor protein (MCP/*CD46*)

CD46 encodes membrane cofactor protein (MCP), a transmembrane complement regulator protein that functions as a cofactor for factor I-mediated cleavage of C3b and C4b (38). MCP is ubiquitously expressed and is particularly abundant on renal cells (39). First described in 2003 in three families (40), variants in *CD46* account for approximately 13% of patients with CM-HUS (20). The majority of these mutations are located in the exon/intronic splice sites encoding the four extracellular SCR domains and lead to loss of MCP surface expression (20, 41). However, in a quarter of cases, MCP expression remains normal, but its function is impaired (41). Patients with *CD46* variants typically have a lower risk of relapse and ESKD.

When present with other complement pathogenic genetic variants, certain *MCP* risk haplotypes, such as *MCP_ggaac,_* may be more likely to relapse and suffer from renal deterioration (42). The *MCP_ggaac_* haplotype is composed of two variants (NM_002389.4 c.989-78G>A and c.*897T>C) that are in non-coding regions, as well as three nucleotides that must be wild-type (c.-652G, c.-366G, and c.1127 + 638A>G). The *MCP_ggaac_* haplotype is common in the general population and not thought to be sufficient to cause disease alone but may increase disease penetrance in individuals with additional pathogenic variants (42, 43).

### 3.2 Gain-of-function variants in complement activating components

Genetic variants in complement-activating proteins result in excessive activation of the complement system and disrupt the balance between activation and regulation (44).

#### 3.2.1 Complement Component 3

C3, the most abundant complement protein, is the central effector molecule of the complement system and forms the C3 and C5 convertases. Structurally, C3 consists of 13 domains and is composed of one α and β chain. It possesses multiple binding sites for ligands and only becomes biologically active upon proteolytic cleavage into C3b.

C3 is cleaved by the C3 convertase to C3b, which is subsequently degraded by factor I in the presence of cofactors such as CFH, MCP, CR1, C4BP, and thrombomodulin (TM). C3b is constantly produced at a very low rate by spontaneous hydrolysis. It can bind to pathogens and host cell surfaces, where it associates with factor B. The C3b-factor B complex is cleaved by complement factor D, generating C3bBb (C3 convertase), which further amplifies C3 activation in a positive feedback loop. This cascade ultimately leads to membrane attack complex (MAC) formation and tissue injury (**Figure 1**).

In CM-HUS, dysregulation of C3 is typically due to variants that lead to gain-of-function of the C3 protein. The C3 variants increase stability or formation of C3 or modify regulatory protein interactions with C3 (45). Most variants are heterozygous, although rare homozygous deletions in the highly conserved thioester-containing domain have been reported. It is hypothesized that these homozygous deletions may impose a conformational change in C3b, making it resistant to inactivation by regulatory proteins (46). The presence of certain *MCP* haplotypes (e.g. as *MCP_ggaac)_* can impact the penetrance of the pathogenic C3 variant and influence the presentation of CM-HUS in an individual.

#### 3.2.2 Complement factor B

Complement factor B is a zymogen that carries the catalytic site of the complement alternative pathway convertases (C3bBb). Upon binding to C3b and cleavage by factor D, factor B participates in the formation of both C3 and C5 convertases, which subsequently cleave their respective substrates, C3 and C5 (**Figure 1**).

Although early studies did not establish an association between *CFB* variants and CM-HUS (47), subsequent research identified a subset of patients harboring gain-of-function mutations in *CFB*. These variants enhance the formation and stability of the C3bBb convertase and/or confer resistance to inactivation by complement regulators (44).

### 3.3 Other non-complement genetic variants associated with CM-HUS

Given that no causative genetic variant is identified in 50% of patients, it is postulated that other, yet undefined, non-complement genetic variants contribute to the pathogenesis of CM-HUS. The high rate of incomplete penetrance in familial CM-HUS also supports the complexity of the underlying genetics, along with the likely contribution of multiple genetic and environmental factors.

Abnormalities in coagulation cascade genes that interact with the complement system, such as *THBD*, *PLG*, and *DGKE*, have been implicated in CM-HUS; however, further studies have called some of these associations into question. Mechanisms for the pathophysiology of *DGKE-*induced TMA remain under investigation but appear to be independent of the complement pathway. Patients with *DGKE* variants are more likely to present in childhood, often with nephrotic syndrome, and are less likely to respond to complement inhibitor therapy (48, 49).

The gene encoding plasminogen, *PLG*, has also been associated with CM-HUS. *PLG* encodes plasminogen, a component of the fibrinolytic pathway. When plasminogen is converted to plasmin, it can cleave complement proteins C3b and C5 and therefore act as a complement inhibitor. The inhibitory effect of plasminogen on complement activation is weak overall, and the association of *PLG* variants with CM-HUS has not been robustly demonstrated (50).

*THBD* was originally associated with CM-HUS; however, more recent data has refuted this association. *THBD* encodes the protein thrombomodulin, which is a transmembrane endothelial cell glycoprotein that has anticoagulation properties but also interacts with complement proteins. Variants in *THBD* were originally associated with CM- HUS (51) in a study where six different heterozygous missense *THBD* variants were identified in 152 patients with CM-HUS. In addition, cultured cells expressing *THBD* variants had decreased capacity to inactivate C3b and to activate procarboxypeptidase B and thus were less protected from activated complement (51). A more recent study investigated 1216 patients with CM-HUS and *THBD* variants were identified in 27 patients. This study highlighted uncertainties regarding *THBD* in the development of CM-HUS. At the time of the original study, large population databases were not available and *THBD* variants were not known to be common in certain ancestral groups. As such, *THBD* variants were found in patients with CM-HUS likely reflecting their prevalence in the general population rather than their involvement in the pathophysiology of CM-HUS. In the more recent study, it was ascertained that most patients with *THBD* variants did not have the characteristic clinical profiles of CM-HUS and tended to resemble secondary HUS (52). Most patients with isolated *THBD* variants had greater than one cause associated with secondary HUS and did not have disease recurrence through follow-up; many tended to recover spontaneously with removal of potential triggers.

## 4 Complement genetic testing and clinical implications

Complement genetic testing utilizes next-generation sequencing (NGS) to perform full gene sequencing and copy number analysis of multiple genes related to the complement system. Sanger sequencing and multiplex ligation–dependent probe amplification (MLPA) may also be performed to confirm findings and evaluate for large deletions (2, 53).

Several centers offer comprehensive complement genetic testing, and the gene panels and turnaround times vary. Multi-gene NGS panels commonly include *CFH, CFI, CD46* (also known as MCP), *CFB, C3*, and *CFHR1-5*. Often, panels may also include non-complement genes, such as *DGKE* and coagulation-related genes. Additionally, *ADAMTS13* may be included due to its association with TTP, which is often in the differential diagnosis. Some panels include the specific *C5* variants that have been associated with eculizumab resistance (30). The most well characterized of these variants are p.Arg885His and p.Arg885Cys polymorphisms. These variants alter the eculizumab binding site on C5 which then prevents complement inhibition with eculizumab necessitating the use of medications that target alternative epitopes of C5 or other complement targets (30).

Laboratories typically use the American College of Medical Genetics and Genomics and the Association for Molecular Pathology (ACMG/AMP) guidelines for variant classification. Clinical classification of genetic variants in CM-HUS remains challenging from a laboratory standpoint for various reasons. The ACMG/AMP criteria were not intended to be used to interpret variants in genes associated with non-Mendelian or multigenic disorders; however, due to a lack of alternative guidelines, the ACMG/AMP has typically been adopted for variant classification in CM-HUS (54). The low penetrance of CM-HUS makes it difficult to establish a minor allele frequency cut-off to identify variants more common in the general population than expected for the disease. Assessment of segregation in family members is not reliable because not all individuals may be exposed to the second trigger. In addition, an individual may have a pathogenic variant but might never develop CM-HUS (55). Many patients will not have pathogenic variants and will present with variants of uncertain significance (VUS). For rare disorders with low penetrance, such as aHUS, it can be difficult to find sufficient evidence to reach a classification of benign/likely benign or pathogenic/likely pathogenic. In a research setting, the variant protein corresponding to genetic variants identified in patients can be expressed *in vitro*, followed by functional studies to characterize the variant protein’s impact on the alternative pathway (16, 56–58). For example, if a complement variant leads to the non-expression of the carrying allele, this would represent strong evidence of pathogenicity. Other useful information to help guide variant classification includes prevalence data of the respective variant (ie demonstrating that the prevalence of the variant in affected individuals is significantly increased compared to the prevalence in controls) (54). When a functional study is performed after a variant has been classified, or when other information emerges (e.g. additional reports of affected individuals with the same variant), a variant classification can be updated, such as to re-classify a VUS as likely benign or likely pathogenic.

Given the nuances of variant classification and genetic test interpretation, it is important for clinical laboratories performing this test to have a deep understanding of this disorder. Paired clinical and genetic data are essential for accurate variant interpretation and meaningful clinical correlation. As the tools available for genetic testing and interpretation become more robust and more data regarding complement variants in CM-HUS emerges previously published incidences of pathogenic variants in CM-HUS will need to be reassessed (16, 22). Due to the complexity of complement genetics and variable penetrance, genetic counseling should be offered to individuals with CM-HUS.

## 5 Factor H autoantibodies

The first published report of Factor H antibodies in CM-HUS in 2005 was a description of three pediatric patients (59). Since then, FH antibodies have become the best-studied acquired drivers of CM- HUS, detected in approximately 24% of pediatric and 19% of adult CM-HUS cases (60, 61). FH antibodies are primarily of the IgG class, and the majority bind to the C-terminal region of FH, impairing its function (59, 62, 63). The C-terminus of FH is needed for FH to be docked onto C3b-covered cells or surfaces, which is a critical step in complement regulation.

FH antibodies are strongly associated with deficiency of FH-related protein 1 (FHR1). Ninety percent of patients with FH antibodies have complete deficiency of the FH-related proteins FHR1 and FHR3 due to a homozygous combined *CFHR3*-*CFHR1* deletion. However, in about 15% of patients, FHR1 deficiency is caused by compound heterozygous deletion of *CFHR3-CFHR1* and *CFHR1-CFHR4* (64). Homozygous *CFHR1-CFHR4* deletion has also been rarely reported (65). In some patients, a heterozygous deletion of *CFHR1*, with or without accompanying deletions in *CFHR3* or *CFHR4*, may be paired with a nonsense or frameshift variant on the other allele. If this combination results in complete absence of CFHR1 protein production, the functional effect may mimic a homozygous deletion (Mayo Clinic, unpublished data). However, this scenario remains poorly characterized, and its clinical significance is not yet fully understood (To date, there have been no reports of Factor H antibodies with this rare clinical situation).

Not all patients with homozygous *CFHR1* and *CFHR3* gene deletion have FH antibodies. In fact, the relative risk for a patient with *CFHR3-CFHR1* deletion developing an FH antibody is small, as homozygous *CFHR3-CFHR1* deletion is common and present in about 3% of Americans of European descent (66). Rates of *CFHR3-CFHR1* deletions vary among populations, as do rates of FH antibodies. European CM-HUS cohorts report FH antibody frequencies of 5-25%, while cohorts in children in India report frequencies in the 50% range (67). Interestingly, the population frequency of the *CFHR1* deletion in healthy cohorts in India was similar to those reported in other studies, suggesting unrecognized mechanisms for the high prevalence of FH-antibody-mediated CM-HUS in Indian children.

Ten percent of patients with FH antibodies do not have a deletion in *CFHR1* (65). In some instances, likely pathogenic variants in *CFH*, *THBD*, and *C3* are enriched in patients who develop FH antibodies (64). A meta-analysis of 19 studies including 384 patients demonstrated a pooled prevalence of pathogenic or likely pathogenic variants of 3% in anti-factor H CM-HUS (68).

Patients with anti-FH CM-HUS are typically younger than those with non-anti-FH CM-HUS (2, 61, 64).

The course of disease presentation is generally more acute with diarrhea and/or fever reported in >50% of cases suggesting that antibody-mediated HUS may be triggered by infection (68, 69). Individuals with high antibody titers at presentation (>8000 U/ml, normal threshold 150 AU/mL) are associated with higher mortality and long-term risk of renal disease (61).

Autoantibodies for Factor I have been described in the setting of CM-HUS but not in C3G. Currently there is no consensus on whether antibodies to FI are definitely implicated in the pathogenesis of CM-HUS. They are exceedingly rare, and thought to form complexes with Factor I, however this seems to have limited impact on complement function. It is still poorly understood how antibodies to FI contribute to disease development, and other susceptibility genetic variants may need to be present (e.g. *CFH*) (70).

*The patient in case 1 had subsequent blood antibody testing that was positive for Salmonella typhi, suggesting an infectious trigger to her acute episode.*

### 5.1 Laboratory assessment of factor H antibodies

ELISA-based assays allow quantification and monitoring of FH autoantibodies (71, 72). The Kidney Disease Improving Global Outcomes (KDIGO) guidelines recommend confirming the presence of FH antibodies in a second sample at least 4 weeks after the initial sample. There are challenges in standardizing the FH antibody assay across laboratories, and this limits the compatibility of antibody titers performed in different laboratories (73) which are best compared in a semi-quantitative scale. While there is no universally defined threshold for antibody titers that confirms anti-FH-related CM-HUS and each laboratory should perform their own reference interval studies, most of Europe has adopted similar cutoffs, with one study reporting that at the time of diagnosis, mean titers associated with CM-HUS were around 5000 AU/mL (range: 2121 -163,829 AU/ml) (60). Following therapy, the antibody titers decreased significantly, with levels dropping below 500 AU/mL by three months of follow-up after treatment, supporting the value of assessing FH antibody levels at diagnosis and during treatment monitoring for CM-HUS (60).

More recently other methods for assessing FH autoantibodies have been explored. For example, an immunochromatographic test (ICT) for detection of factor FH autoantibodies free or bound to FH was developed (74). However, these assays are not yet ready for use in clinical practice as they are not commercially available.

## 6 Functional assessment of complement abnormalities

### 6.1 Complement Serology to assess fluid phase markers of complement

CM-HUS is expected to have abnormal serum markers of the alternative pathway of complement, while the classical pathway components should be within reference range. As such, the expected serum complement profile would have low C3 and normal C4. Only assessing C3 and C4 offers low sensitivity; therefore, attempts to develop a more comprehensive panel assessment with complement components and complement activation products have been undertaken with the hope that a broader assessment would offer more diagnostic utility. Different laboratories offer different panels of complement analytes and single-test options.

A systematic review of 60 studies published through 2021 demonstrated that patients with CM-HUS have lower C3, CH50, AH50, and CFB compared to the reference range. C5a, C5b-9, and Bb were higher among patients with CM-HUS compared to the reference range, while C4, C4d, CFH, and CFI were more likely to be within reference range in patients with CM-HUS (75). Complement serology is most diagnostically useful when obtained prior to initiation of plasma exchange and use of complement inhibitor therapy. After receiving complement inhibitor therapy, the diagnostic utility of complement component serology is questionable. CH50 and AH50 functional tests are decreased or undetectable because eculizumab blocks recruitment of C5, which is a necessary step for these functional tests (76).

### 6.2 Cell-directed complement activation evaluation

C5b-9 is sometimes monitored after starting treatment with complement inhibitor therapy, however, there is no standardized consensus on this, and approaches vary based on practice preference. Even though C5b9 formation is blocked when patients use C5 inhibitors, the test is not especially useful to aid in monitoring eculizumab complement blockage. Complement activity upstream is recommended for that application instead (CH50 or AH50 tests, or C5 function). Normal sC5b-9 does not rule out residual complement disease, and mild elevations may be observed even in clinical remission stages of therapy. SC5b-9 concentrations are not associated with C5 inhibitor success or efficacy, but may be used as means to trend patients over time if the baseline test is abnormal (76).

Compared to complement component tests and complement activation products evaluation, cell-directed complement activation is proposed to be more specific for CM-HUS diagnosis. Two ex-vivo, cell-directed methodologies include microvascular endothelial cell (HMEC-1) staining for C5b-9 and the modified Ham (mHam), which evaluates complement-induced cytotoxicity.

HMEC uses indirect immunofluorescence microscopy to assess for increased C5b-9 deposition in wild-type HMEC-1 endothelial cells incubated with patient serum stimulated by adenosine diphosphate (77, 78). One of the barriers to incorporating ex-vivo, cell-directed complement assessment into the diagnostic algorithm for CM-HUS has been the lack of commercially available assays. More recently, the mHAM assay has become commercially available. This bioluminescence assay uses a CD46 knockout HEK293 cell line and measures complement activation in patient serum by comparing bioluminescence reduction to baseline (heat-inactivated patient serum). Bioluminescence indicates cell viability; therefore, decreased bioluminescence is a surrogate for complement-mediated cell death. Positive patient serum samples by this assay are reflexed to a second study after addition of a complement inhibitor to confirm complement specificity (79, 80). Although an exciting option to help with diagnosis of CM-HUS, further clinical validation and its use in real world practice is necessary.

*In case 1, laboratory data in support of CM-HUS included a positive factor H antibody and the associated CFHR3-CFHR1 deletion. Case 2 had complement serology demonstrating complement activation, with suspicion for alternative pathway involvement given normal C4. The findings of no complement variant did not support or refute a diagnosis of CM-TMA.*

In addition to its usefulness in CM-HUS diagnosis, complement testing (genetics and serology assessment) also has a role in complement inhibitor therapy duration decisions. Nuances regarding implications of complement testing on treatment are discussed below.

## 7 Treatment

Effective management of CM-HUS requires a multidisciplinary approach, with coordinated input from hematologists, nephrologists, pathologists, and geneticists to guide diagnosis and treatment.

**Figure 3:**
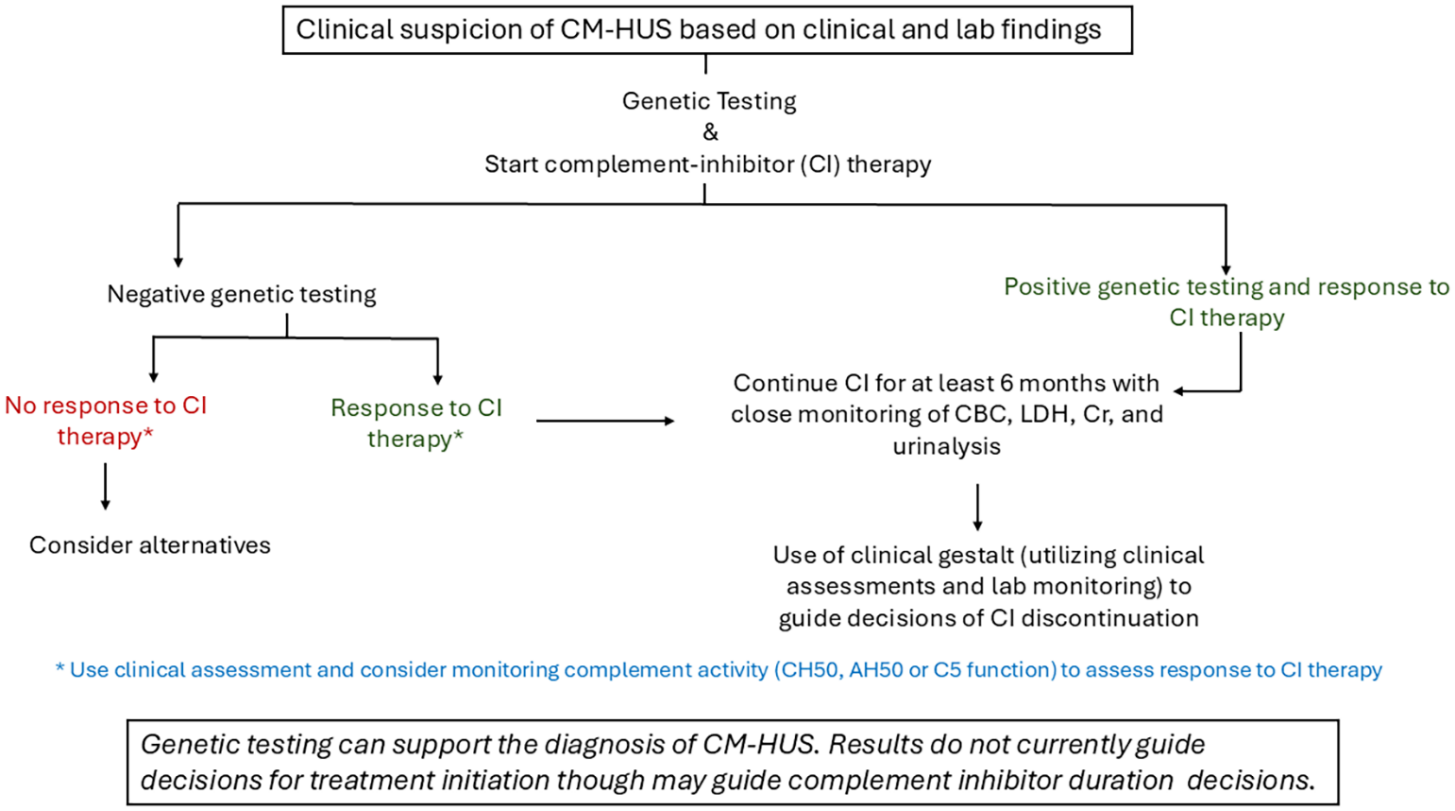
General treatment approach for non- antibody associated CM-HUS.

Management of CM-HUS is guided by the underlying pathophysiology. In cases of non-antibody-associated CM-HUS treatment is C5 inhibitor therapy (**Figure 3**) while in FH-antibody-associated- CM-HUS, the goal is antibody elimination. There are two FDA-approved C5 inhibitors for pediatric and adult patients with CM-HUS: eculizumab and ravulizumab. Prior to complement inhibitor therapy, management was supportive care with or without plasma exchange (PEX). Response to PEX is variable and dependent on which complement protein/genetic variant is implicated and, in many instances, can be suboptimal. In patients with MCP variants, plasma-based therapy is not thought to have a great impact on CM-HUS outcome; one potential explanation being that since MCP is a membrane-bound protein, plasma infusion or exchange would not fix the defect. In contrast, patients with CFH variants treated with plasma may have a response, but not consistently (20). In contrast, patients with Factor H antibodies do respond to PEX.

Eculizumab, approved in 2011, is a humanized recombinant monoclonal IgG antibody that binds to and inhibits C5. It effectively treats CM-HUS and has the potential to rescue renal function. Since eculizumab’s approval, real-world data have continued to demonstrate its efficacy. The largest single retrospective comparative cohort study reported efficacy data of patients treated with eculizumab (n=192) compared to a historical control cohort (n=279) from before availability of eculizumab. The 5-year cumulative estimate of ESKD-free survival was 39.5% in the control cohort and 85.5% in the eculizumab-treated cohort with a mutation or Factor H autoantibodies (p=0.000) (23). Prognosis varied according to mutation type in the control cohort. Five-year ESKD-free survival was worse in those with *CFH*, *CFHR1:CFH* hybrid, *CFI*, and *C3* variants compared to those with *CD46* variants or FHAA. For those with a *CFH*, *CFI*, and *C3* mutation, ESKD-free survival was significantly better in the eculizumab-treated group, while individuals with a *CD46* mutation did not have improved ESKD-free survival in the eculizumab-treated group. There was also no statistically significant difference in ESKD-free survival in individuals with FH-antibody-mediated CM-HUS.

Ravulizumab, FDA approved in 2019, was engineered from eculizumab to have extended complement inhibition with a 4 amino acid difference in its heavy chain. In a phase 3 trial, complete CM-HUS response was observed in 53.5% of patients treated with ravulizumab (median time to response of 86 days (81). There has been no head-to-head comparison between eculizumab and ravulizumab, but comparison studies of patients within the clinical trials have been performed. One study used propensity scoring to compare patient-level data from four eculizumab clinical trials (n=80) and two ravulizumab clinical trials (n=79). At 26 weeks, renal dysfunction, hematologic parameters, and dialysis prevalence were improved with both eculizumab and ravulizumab. Fatigue and quality of life were also improved. There were no statistically significant differences in outcomes between the two different complement inhibitors; however, confidence intervals were wide (82).

### 7.1 Complement inhibitor duration

There is no question that complement inhibitor therapy has led to significant improvements in morbidity and mortality and changed the natural history of the disease for patients with CM-HUS. The duration of complement inhibitor therapy remains a subject of ongoing investigation. There is no evidence to support lifelong therapy in all patients with CM-HUS, and safely discontinuing complement inhibitor therapy in a select cohort of patients has been reported. A systematic review based on studies published before January 2021 identified 280 cases of eculizumab discontinuation, with relapse occurring in 83 (29.6%) of cases. Relapse rates were highest among patients with variants in *CFH* and *MCP/CD46.* Presence of a renal allograft, decreasing age, and detection of variants in *CFH*, *MCP/CD46*, or *C3* were all independently associated with relapse after eculizumab discontinuation. One unique finding in this study was the higher risk of relapse in patients with *CD46* variants; however, relapse in this subgroup was primarily driven by patients with variants within canonical splice sites (94). Some postulates regarding this finding include decreased complement inhibitor therapy exposure due to presumption of a more ‘benign’ course. Another hypothesis is that because historically variant classification has not been standardized, it is possible that *MCP/CD46* has been associated with a more benign course because the common MCP_ggaac_ risk haplotype was being grouped together with, and considered the same as, other pathogenic variants.

A large country-wide (UK) cohort study published after the systematic review demonstrated a relapse rate of 1 per 9.5 person years without eculizumab for those with a pathogenic mutation and 1 per 10.8 person-years without eculizumab for those with a VUS. There was no relapse in 67.3 person-years in those without a genetic variant in a complement gene (23).

The compilation of available data supports the consideration of complement inhibitor discontinuation in patients considered to be low risk for relapse.

### 7.2 Risks of complement inhibitor therapy

Complement inhibitor therapy is typically well tolerated in most patients. Reported adverse events include infections (upper respiratory tract and urinary tract), hypertension, diarrhea, arthralgia, headache, and fever.

Due to an increased risk of meningococcal infection with complement inhibitor therapy, the FDA has issued a boxed warning regarding the risk of serious meningococcal infection. This warning advises vaccination against *Neisseria meningitis* two weeks prior to the start of complement inhibitor therapy.

Prescribers must also be part of a Risk Evaluation and Mitigation Strategy (REMS) program (FDA). Penicillin V potassium, ciprofloxacin, or levofloxacin is required if a patient needs to start complement inhibitor therapy immediately. Due to incomplete protection from vaccines alone, the Centers Disease and Control (CDC) recommends continued antibiotic prophylaxis for as long as an individual remains on complement inhibitor therapy (83).

### 7.3 Management of FH- antibody -associated CM-HUS

In cases of FH-antibody-associated-CM-HUS, the goal is antibody elimination with two methodologies: PEX or immunosuppressive therapy. PEX effectively reduces anti-FH autoantibody titers, with lower titers correlating with improved outcomes (60, 84, 85). However, no standardized target titer has been established, though high titers such as ≥1300 AU/mL at six months post-treatment have been linked to higher relapse rates (84).Options for immunosuppression include corticosteroids, cyclophosphamide, and rituximab. These therapies can be combined with PEX (85).

Relapse rates of anti-FH associated CM-HUS treated with PEX and immunosuppression range from 11% to 75%, with relapse occurring within the first one to two years. High FH antibody titers and low free Factor H predispose to risk of relapse.

There is limited prospective data evaluating complement inhibitor therapy for FH-antibody-associated CM-HUS; however, several retrospective small cohort studies demonstrated efficacy, with all patients treated with eculizumab achieving a remission. Some patients received eculizumab with immunosuppression and PEX, while others received no immunosuppression (86) A recent study demonstrated outcomes of patients with FH antibodies who also received eculizumab along with immunosuppression. Eculizumab was safe and effective in inducing remission in all 10 patients. The authors concluded that long-term treatment with eculizumab and mycophenolate mofetil (MMF) was sufficient therapy for mild FH-antibody-associated CM-HUS and could replace PEX. In FH-antibody-associated CM-HUS, the titer at which to discontinue eculizumab has not been established. In a prospective multicenter open-label study that included four patients with FH-antibody-associated CM-HUS, eculizumab was discontinued when antibody titers decreased to 473–1500 AU/mL (compared to active disease titers of 9000–60000 AU/mL) (87). There were no relapses over a 2-year follow-up period. Fakhouri et al. recently recommend discontinuing complement inhibitor therapy when FH-antibody titers are less than 1000 AU/mL (53).

*In Case 1, immunosuppression was discontinued with improvement in FH antibodies, but eculizumab was continued due to a variant likely impacting CFH function. In-silico modeling tools were used to guide this decision. In Case 2, the decision was made to discontinue eculizumab after six months of therapy, given the overall lower risk of relapse with no complement variants identified.*

### 7.4 Long term outcomes and renal transplant

Before the use of eculizumab, 50% of patients progressed to ESKD with their first episode (6, 20). Due to high risk of recurrent disease (reported in 60-80% of patients with a prior diagnosis of CM-HUS undergoing transplant), patients with CM-HUS were largely excluded from receiving a renal transplant, as recurrence is associated with transplant failure. The risk of post-transplant relapse is driven by the underlying complement abnormality. Patients with complement gene variants in *CFH*, *CFI*, *CFB*, and *C3* have a higher risk of relapse. Studies have demonstrated that use of eculizumab in the peri-transplant and post-transplant periods reduces the risk of relapse (88). The 2016 KDIGO guidelines recommend prophylactic eculizumab in patients at moderate or high risk of recurrence, with risk of recurrence guided by the presence and type of complement variants (**Table 1**) (93). Due to the cost of eculizumab, some countries do not offer prophylaxis but instead use on-demand treatment for patients with relapse. The Dutch CUREiHUS study reported outcomes of on-demand eculizumab treatment in a cohort of patients preferentially receiving a living donor kidney transplant and treated with an endothelial protective regimen post-transplant. A comparative analysis of patients from the UK treated with prophylactic eculizumab versus the Dutch cohort demonstrated that death-censored graft survival was not inferior with rescue or on-demand eculizumab compared to prophylactic eculizumab (89). However, until larger studies are available, if eculizumab is accessible, the general practice at most centers is to consider eculizumab prophylaxis per KDIGO guidelines.

## 8 Future insights

Efforts to standardize variant classification in multigenic and incompletely penetrant disorders such as CM-HUS are ongoing and critically needed. While the ACMG/AMP guidelines provide a foundational framework for variant interpretation, they were originally designed for Mendelian disorders and may not fully capture the complexity of oligogenic and multifactorial diseases. Initiatives from different groups have begun to address these gaps. In complement-mediated diseases, statistical modeling and large-scale functional assays have helped reclassify variants in genes such as CFH, moving the field closer to more accurate, consistent, and clinically meaningful genetic interpretation. These developments will help improve genotype-phenotype correlation in CM-HUS (16, 90–92). In addition, as larger data sets have become available it may be possible to begin to understand the interplay among common variants that may modestly impact protein function but together, along with environmental factors, contribute to disease presentation even in the absence of rare pathogenic variants.

## 9 Conclusion

CM-HUS reflects the evolving intersection of complement dysregulation, genetic predisposition, and clinical variability. C5 inhibitors have changed the natural history of the disorder, however many questions regarding optimal duration and use in the setting of renal transplant remain. The growing recognition of overlapping genetic variants, incomplete penetrance, and antibody-mediated phenotypes continues to challenge traditional frameworks of diagnosis and treatment and push us toward more individualized strategies. As diagnostics improve and real-world data expand, we are moving towards greater precision, matching the right treatment strategy to each patient, guided by molecular insight and clinical judgement.
